# Construct Validity of the Pictogram Test for Diagnosis of Schizophrenia

**DOI:** 10.7759/cureus.21383

**Published:** 2022-01-18

**Authors:** Luba Leontieva, Sergey Golovko

**Affiliations:** 1 Psychiatry, State University of New York Upstate Medical University, Syracuse, USA; 2 Psychiatry, Hutchings Psychiatric Center, Syracuse, USA

**Keywords:** cross-cultural studies, pictogram test, mmpi-2, construct validity, schizophrenia and other psychotic disorders

## Abstract

Objectives: To examine construct validity of the Pictogram Test (PT) which assesses disturbances in thinking in individuals with schizophrenia. The PT was developed in Russia; it was found to be applicable for the English-speaking population of the USA. The variables of the PT were correlated with Minnesota Multiphasic Personality Inventory (MMPI-2).

Method: Russian-and English-speaking participants completed the PT and MMPI-2 in their native languages.

Results: The PT variables that reflected attribute selection choice of intermediate concepts for memorization and the variables that were geometrical shapes had significant correlations with MMPI-2 scales linked to schizophrenia. This represents evidence of convergent validity. The same PT indices did not significantly correlate with most of the MMPI-2 scales that are not elevated in schizophrenia, representing some discriminant validity of the PT.

Conclusions: The less often the participants were able to connect target words with economical intermediate concepts, the higher were the elevations of schizophrenia-related scales. Also, the more abstract and remote their intermediate concepts, the less often they recalled targets. These findings give evidence of the validity of the PT in assessing the thinking of individuals with schizophrenia and related conditions.

## Introduction

Thinking distortions and schizophrenia

Cognitive disturbances and disordered associations in schizophrenia were outlined by the forefathers of modern psychiatry Eugen Bleuler [1] and Emil Kraepelin [2]. Now, over 100 years later, there are many studies that prove the concept that cognitive deficits precede the development of positive symptoms of schizophrenia (i.e. hallucinations and delusions) [3-5]. In his recent article, Kahn outlined the path to new studies in the area of investigating cognition in early adolescence, including using biomarkers, brain imaging, and genetics to understand better the causes of schizophrenia [6]. Finding new and sensitive psychological tests that assess cognition might be a helpful tool for such new studies. Logical memory has many indirect definitions in the literature. The main theme of these definitions is that logical memory involves memory storage and consolidation, which in turn involves meaning. Lezak et al. [7] and Squire [8] described that patients with damage to the medial temporal limbic and the midline diencephalon areas are more likely to have significant memory storage and consolidation problems. Due to their structural brain damage, these patients cannot assign meaning to the materials needed to be remembered. Recent research found that patients with schizophrenia have abnormal brain connectivity in the same areas that precede the development of positive symptoms [9,10].

Developing psychological testing that could tap into associative or logical memorization could aid the early diagnoses of those vulnerable to developing schizophrenia. As logical thinking is problematic in individuals with schizophrenia, such test results could help in the evaluation of treatment efficacy as well as the differential diagnosis of schizophrenia from other psychotic disorders. The most commonly used logical memory test is the Logical Memory (LM) subtest of Wechsler Memory Scale-IV [11]. However, the LM test does not give us information on the mechanism of memorization. The Pictogram Test (PT) taps into associative memory and the meaning the test-taker attaches to the materials needed to be memorized and later retrieved. The construct validity of the PT is investigated in this study by the means of convergent and discriminant validation of the three PT indices with Minnesota Multiphasic Personality Inventory 2nd edition (MMPI-2) scales [12]. Convergent validity was examined by correlating PT indices with MMPI-2 scales that are associated with schizophrenia: Paranoia (Pa), Schizophrenia (Sc), Bizarre Mentation (BIZ), Bizarre Sensory Experiences (Sc6,) and Persecutory Ideas (Pa1). Discriminant validity was examined by the PT indices correlation with MMPI-2 scales elevation, which does not necessarily signify schizophrenia: Hypochondriasis (Hs), Depression (D), Hysteria (Hy), Psychopathic Deviate (Pd), Masculinity-Femininity (Mf), Psychasthenia (Pt), Hypomania (Ms), and Ego Strength (Es).

Brief validity overview

Construct validity is one of the main psychometric properties that reflect a test’s ability to measure what it intends to measure. Two of the several sources of evidence of the presence of construct validity are convergent and discriminant validation procedures. Convergent validity shows that a certain test correlates highly with another test that measures the same behavioral/psychological domain. If the test variables measure the same behavioral or psychological domain as the other test variables measure, it is possible to conclude that this test is a valid estimation of specific behavior governed by this psychological domain. Discriminant validity shows that a certain test does not correlate with tests that measure different behaviors.

## Materials and methods

The project was approved by the West Virginia University School of Medicine Institutional Review Board (IRB) with approval number 15795-B for English-speaking subjects and Ethics Committee of the 3rd Psychiatric Hospital in St. Petersburg, Russia, with approval number 942 for Russian-speaking subjects and was in accordance with requirements as outlined in the Declaration of Helsinki.

Data collection

A detailed description of the study participants and method of recruitment is outlined elsewhere [13]. Briefly, the data were collected between 2002-2003, half of the participants were English-speaking from the USA while the other half were Russian-speaking from Russia. There were one-half of inpatients diagnosed with schizophrenia and schizoaffective disorder and one-half normal, control participants in each language group.

Inclusion and exclusion criteria

The diagnosis of schizophrenia in the patients’ charts was the criterion for inclusion. We did not include patients who were too unstable or disorganized to complete the assessment. Examples of these were patients actively hallucinating, did not understand the tasks, did not follow instructions, aggressive and hostile. No specific scales for exclusion criteria were administered.

Measures

The Beck Depression Inventory-II (BDI-II) [14], MMPI-2, and PT were the measures used in the original study. For the purpose of the present report, only MMPI-2 scales of interest and the PT data are analyzed. All measures were administered in the participants’ native language (Russian or English).

Pictogram test

The detailed description of the PT [15] and its cross-cultural applicability is described elsewhere [13]. Briefly, the PT involves memorization of 16 words and phrases that vary in their degree of abstraction by using examinees’ own associative drawings that are supposed to have meaning for the examinees' connections to the words/phrases to be memorized. The PT assessment form is included in the appendices section of this article. 

The artistic quality of drawings was not judged. The PT was found applicable for an English-speaking population and found to have high inter-rater reliability and internal consistency [13]. The same study conducted the correlational analysis of PT variables, which allowed examination of the variables in relationship to each other; the number of variables were combined into statistically manageable and conceptually meaningful indices. These indices reflect participants’ use of intermediate concepts to memorize abstract materials. The indices are: Concrete Index (CI), Attribute Index (AI), and Geometric Index (GI). CI contains photographic-like drawings of people, usually engaged in some activity. Such drawings may be cumbersome and may or may not be helpful in retrieval. AI contains more concise drawings selected according to the principle of belonging (i.e., Christmas tree for “happy holidays”) and is helpful in retrieval. GI is characterized by meaningless/vacuous drawings and is very rarely helpful in retrieval. A previous study revealed that AI was the strongest discriminator, correctly classifying 91% of English-speaking and 86% of Russian-speaking participants as either patients or non-patients, as cited in [13]. This indicates the importance of this PT index when assessing logical thinking disturbance in schizophrenia. Patients with schizophrenia lack the ability to form economic and logically connected intermediate concepts for the productive memorization of abstract materials in their PTs (low AI). GI was also significant in its separation of patients from non-patients. Patients with schizophrenia tended to use geometric forms as mediators, which did not help them with memorization. CI did not discriminate patients from non-patients in the prior study [13].

Minnesota multiphasic personality inventory (MMPI-2)

MMPI-2 [12] test represents a 567-item self-administered yes/no questionnaire. All participants completed all 567 questions; for some patients from the schizophrenia group the questions were read by the examiner and the administration was divided into parts due to fatigue. For Russian participants, the research MMPI-2 Russian version was used [12,16]. The following scales were used for convergent validity: clinical scales 6 (Pa = Paranoia), 8 (Sc = Schizophrenia), content scale BIZ (Bizarre Mentation), supplementary scale Sc6 (Bizarre Sensory Experiences) and Harris-Lingoes subscale Pa1 = Persecutory Ideas. Test-retest reliability of MMPI-2 scales Pa, Sc, BIZ, Sc6, and Pa1 for diagnoses of schizophrenia ranges from 0.58 to 0.89; internal consistency ranges from 0.34 to 0.87. These scales have been found to be associated with the psychiatric diagnosis of schizophrenia, thus showing criterion-related validity [17,18]. The following scales that are not necessarily and consistently elevated in schizophrenia spectrum disorders were used for discriminant validity: (1) Hypochondriasis (Hs); (2) Depression (D); (3) Hysteria (Hy); (4) Psychopathic Deviate (Pd); (5) Masculinity-Femininity (Mf); (7) Psychasthenia (Pt); (9) Hypomania (Ms); and supplementary scale Ego Strength (Es). 

We excluded MMPI-2 protocols that had elevated validity scales suggesting invalidation of the test responses. Specifically, we excluded those protocols that had scales L higher than 65, F higher than 100, and K higher than 65. This left us with a total of 87 protocols. Thus, the correlational analysis included 87 MMPI-2 and Pictogram Test protocols, both from patients and non-patients. 

Research design, hypotheses, and statistical data analysis

A correlational analysis was used for the present paper. Three PT indices (CI, AI, and GI) were correlated with MMPI-2 scales Pa, Sc, BIZ, Pa1, and Sc6 for convergent validity and with scales 1, 2, 3, 4, 5, 7, 9, 0, and Es for discriminant validity. Pearson correlation coefficients of 0.3 and above were considered as reflective of commonly shared variances of MMPI-2 scales and the PT indices. In the statistical analysis of the data, a hypothesis that a correlation coefficient between two variables is significantly different from zero was tested (p < .05). The practical significance of the correlation coefficient was interpreted. In this study, we used patient and non-patient data to increase the total number of protocols used in the correlational analysis. Adjusted p values are calculated from Benjamini and Hochberg method to control the false discovery rate given multiple comparisons [19].

## Results

A total of 142 participants were involved in the original cross-sectional study; 72 were native Russian speakers in Russia and 70 were native English speakers in the United States. Eighty-seven cases were selected for the correlational analysis. The socio-demographic and clinical characteristics of these participants are summarized in Table 1.

**Table 1:** Socio-demographic and clinical description of study participants who gave valid MMPI-2 (N= 87) § Ukrainian, Byelorussian, Armenian, Korean, Tatar, Bashkir (for Russian-speaking; primary language was Russian). Indian, Turkish, Pakistani, Brazilian, Venezuelan (for English-speaking; primary language was English) ¶ Sertaline, fluvoxamine, paroxetine, fluoxetine, and escitalopram, venlafaxine XR, mirtazapine, trazadone, and clomipramine £ Gabapentin, oxcarbazepine, divalproex sodium, topiramate, lithium

	Non- Patients					Patients		
	Russian Speaking(N=30)				English Speaking (N=26)	Russian Speaking (N=14)	English Speaking (N=17)	
	N (%)				N (%)	N (%)	N (%)	
Sex:								
Male	15 (50%)				18 (46%)	4 (29%)	13 (77%)	
Female	20 (49%)				21 (54%)	10 (71%)	4 (23%)	
Nationality/Ethnicity:								
Russian		35 (85%)			-	14 (100%)		-
Other^§^		6 (15%)			9 (26%)	2 (6%)		
Caucasian		-			22 (69%)	-		16 (94%)
African-American		-			2 (5%)	-		1 (6%)
Students		16 (39%)			33 (85%)	-		2 (12%)
Schizophrenia type:								
Paranoid						13 (93%)		11 (65%)
Simple/undifferentiated/catatonic						-		2 (12%)
Schizoaffective disorder						1 (7%)		5 (29%)
Psychiatric medications:								
Antipsychotics:								
Typical		-			-	12 (39%)		4 (13%)
Atypical		-			-	4 (13%)		24 (77%)
Both		-			-	15 (48%)		3 (10%)
Antidepressants ^¶^		-			-	2 (6%)		10 (32%)
Mood stabilizers^£^		-			-	0 (0%)		9 (29%)
Anxiolytics		-			-	10 (32%)		3 (10%)
Anti-parkinsonian		-			-	10 (32%)		2 (6%)
Anti-seizures		-			-	0 (0%)		1 (3%)
History of alcohol abuse/dependence						2 (14%)		11 (36%)
			Mean (SD)	Mean (SD)		Mean (SD)		Mean (SD)
Age			27 (9)	28 (7)		37 (8)		37 (10)
Duration of illness (in years)			-	-		14 (9)		14 (9)
Education (years)			14 (2)	18 (2)		13 (2)		13 (3)
Number of hospitalizations			-	-		9 (6)		8 (8)

Correlations

Convergent Validity

AI and GI had significant correlations with all MMPI-2 scales of interest. Specifically, the AI had significantly high negative correlations with all MMPI-2 scales of interest: Pa, Sc, Pa1, and Sc6; and a significant moderate to high negative correlation with BIZ. These correlations indicate that the higher the T-scores on these scales, the lower the AI scores. The GI had significant moderate positive correlations with MMPI-2 scales Pa, Sc, BIZ, and Sc6, and significant positive low to moderate correlation with Pa1. These correlations indicate that the higher the T-scores on these scales, the higher the GI scores. The CI did not correlate significantly with any of the MMPI-2 scales of interest. 

Discriminant Validity

AI had a low moderate negative correlation with scales Hs, D, Mf, Pt, and Si. These correlations mean that the higher the T-scores on these scales, the lower the AI. AI has a highly significant positive correlation with Es. GI had significantly low positive correlations with MMPI-2 scales Hs, D, Hy, Pt, and a highly significant negative correlation with Es. CI had only a weak significant positive correlation with scale Mf. See Table 2 for correlational analysis.

**Table 2:** Pearson’s correlations between AI, GI, and CI and MMPI-2 scales (N=87) ** p< 0.01 level (2-tailed). * p< 0.05 level (2-tailed). AI – Attribute selection index; GI – Geometrical index; CI – Concrete index; Pa – Paranoia; Sc – Schizophrenia; BIZ – Bizarre mentation (Content scale); Pa1 – Persecutory ideas (Harris-Lingoes scale); Sc6 – Bizarre sensory (Harris-Lingoes scale). Hypochondriasis (Hs), Depression (D), Hysteria (Hy), Psychopathic Deviate (Pd), Masculinity-Femininity (Mf), Psychasthenia (Pt), Hypomania (Ms), Social Introversion (Si), and Ego Strength (Es).

	Pa	Sc	BIZ	Pa1	Sc6	Hs	D	Hy	Pd	Mf	Pt	Ma	Si	Es
AI	-.52**	-.52**	-.47**	-.50**	-.55**	-.30*	-.38**	-.19	- .08	-.23	-.39**	-.13	-.41**	.58**
GI	.37**	.32*	.38**	.24*	.35**	.26*	.37**	.30**	.08	.08	.25*	.02	.31**	-.44**
CI	.12	.11	.02	.16	.17	.04	-.01	-.04	-.09	.26*	.07	.19	-.01	-.09

## Discussion

This study tested the construct validity of the PT by examining convergent and discriminant validities of the indices created from the PT variables with MMPI-2 scales. The construct validity is the most important psychometric property of any psychological test as it proves that the test is measuring what it is supposed to measure [20]. Our results demonstrate that the AI had the strongest significant correlations with MMPI-2 scales indicative of schizophrenia spectrum illnesses: Paranoia, Schizophrenia, Bizarre Mentation, Persecutory Ideas, and Bizarre Sensory. Specifically, the higher the elevation on these scales, the less likely the drawings were succinct and economical and the less they were chosen based on principles of belonging. In other words, patients with schizophrenia or schizoaffective disorder rarely created intermediate drawings that were “items” (i.e., shovel for “hard work” or Christmas tree for “happy holiday” or thermometer for “illness”) which could be helpful for memorization and recall. Instead, they frequently drew unrelated geometrical shapes that did not help them in recall (GI). GI had a significant positive correlation with scales Pa, BIZ, and Sc6. Thus, both AI and GI correlations with MMPI-2 scales indicative of schizophrenia give evidence of convergent validity of these PT indices. 

We examined the discriminant validity of the PT indices. We selected the MMPI-2 scales that are non-necessarily indicative of schizophrenia. The AI does not have significant correlations with scales Hy, Pd, Mf, and Ma, which give evidence of discriminant validity of AI. Results indicated that AI does correlate significantly, weakly, and negatively with scales Hs, D, Pt, and Si. These negative correlations could be explained by the fact that those who have schizophrenia/schizoaffective disorder could commonly be depressed (D elevation) or/and socially isolated (Si elevation) and have many somatic complaints (Hs and Pt elevation). These symptoms may be associated with impoverished thinking and less available economical intermediate concepts with subsequent poor recall (low AI). GI had no significant correlation with scales Pd, Mf, and Ma. GI had significant weak positive correlations with Hs, D, Hy, Pt, and Si. These correlations could be explained by difficulties in people with psychosomatic symptoms and depression to select economic intermediate concepts for memorization. Thus, GI had shown some evidence of discriminant validity as it does not significantly correlate with scales that do not necessarily signify schizophrenia spectrum illness (Pd, Mf, Ma).

The correlation of the PT incises with Ego Strength (Es) scale warrants the discussion. The high Es scale signifies that the test-taker has good cognitive and emotional reserves, perseverance, self-confidence, intelligence and good reality testing. Our results revealed that AI had a strong significant positive correlation with Es. Specifically, the higher the AI, the higher the Es. This means that subjects with good reality testing have more organized intermediate concepts for memorization. On the other hand, the GI has a strong significant negative correlation with Es. This means that the more disorganized the intermediate concepts for memorization, the lower the Es. Both indices' correlations with the Es scale give evidence of actual conversion validity of the findings, opposed to the initial hypothesis of the discriminant validity. 

Recently proposed theory on the linguistics of schizophrenia and thought disorder associated with it states that positive symptoms and thought disorganization fall into place as failures in language-mediated forms of meaning [21]. Associated memory with the intermediate concepts that the PT taps into shows exactly the meaning the test-takers attach to memorize the abstract concepts. The finding of the current study that PT has a construct validity with MMPI scales associated with schizophrenia gives evidence of the concept that the PT measures. This concept is that associative memory is deficient in patients diagnosed with schizophrenia and related illnesses. 

The CI index did not show significant correlations with any MMPI-2 scales except Mf (moderate positive correlation). This could be an incidental finding. Thus, the predominance of concrete intermediate concepts (i.e., a scene with people) although not ideal and economical for memorization, did not signify schizophrenia spectrum illness.

Limitations

We have no data on the severity of schizophrenia symptoms measured by the standardized instruments. There is a chance that co-morbid disorders such as traumatic brain injury or intellectual/learning disability contribute to some patients' performance. We have no explanation at this point of why CI had mild significant correlations with Mf scales (measures of masculinity-femininity). There is a small chance that individuals who have more concrete drawings (CI) had disturbances in memory as well, which prevents them from having more efficient memorization.

## Conclusions

In summary, the present analysis indicates that two PT indices, AI and GI, correlated with some relevant MMPI-2 scales and, thus, provide evidence for the PT’s convergent and discriminant validity. Both of these validities give evidence for construct validity of the PT with MMPI-2 scales elevated in schizophrenia. With the evidence of the construct validity of the PT, this test could be added to psychological assessment batteries when the clinical question of schizophrenia diagnosis is being raised. Such addition could give an incremental validity to the result of the test battery and, as a result, more diagnostic certainty. With more diagnostic certainty and accuracy, more targeted treatment and remediation of schizophrenia could be designed. 

## Appendices

**Table 3:** PT assessment form Other details- Date______________________________________ Name_____________________________________ Gender____________________________________ Age_______________________________________ Nationality (race)____________________________ Education__________________________________ Have you been tested before? ___________When?_____________ Duration of psychiatric illness_______________________________ Place of the current testing _________________________________

#	WORD/PHRASE	Drawing Time	What is depicted	Explanation of the drawing	Retrieval
1	Happy Holiday				
2	Hard Work				
3	Good Dinner				
4	Illness				
5	Sadness				
6	Happiness				
7	Love				
8	Development				
9	Separation				
10	Deception				
11	Victory				
12	An Act of Heroism				
13	Animosity (enmity, feud)				
14	Fairness (justice)				
15	Doubt				
16	Friendship				
